# Inter-Breath-Hold Geometric and Dosimetric Variations in Organs at Risk during Pancreatic Stereotactic Body Radiotherapy: Implications for Adaptive Radiation Therapy

**DOI:** 10.3390/cancers15174332

**Published:** 2023-08-30

**Authors:** Hamed Hooshangnejad, Devin Miles, Colin Hill, Amol Narang, Kai Ding, Sarah Han-Oh

**Affiliations:** 1 Department of Biomedical Engineering, Johns Hopkins University, Baltimore, MD 21287, USA; 2 Department of Radiation Oncology and Molecular Radiation Sciences, Johns Hopkins University, Baltimore, MD 21287, USA; dmiles18@jhmi.edu (D.M.); colin.hill@nyulangone.org (C.H.); anarang2@jhmi.edu (A.N.); kding1@jhmi.edu (K.D.)

**Keywords:** pancreatic cancer radiotherapy, inter-breath-hold anatomical variability, adaptive radiation therapy

## Abstract

**Simple Summary:**

Deep-inspiration breath-hold (DIBH) techniques are widely used for motion management during CT simulation and treatment delivery, with the underlying assumption of minimal inter-breath-hold variation in organ-at-risk (OAR) geometry. Using a unique dataset from 20 patients, this study presents a novel investigation of the OAR geometric variation between multiple DIBH CT scans in pancreatic cancer stereotactic body radiotherapy (SBRT), and discusses implications for plan accuracy and OAR toxicity. We demonstrated significant dosimetric variation in abdominal OARs during pancreatic SBRT from inter-breath-hold geometric variation. Modern adaptive radiation therapy (ART) techniques with real-time OAR tracking may be able to reduce these uncertainties.

**Abstract:**

Pancreatic cancer is the fourth leading cause of cancer-related death, with nearly 60,000 cases each year and less than a 10% 5-year overall survival rate. Radiation therapy (RT) is highly beneficial as a local-regional anticancer treatment. As anatomical variation is of great concern, motion management techniques, such as DIBH, are commonly used to minimize OARs toxicities; however, the variability between DIBHs has not been well studied. Here, we present an unprecedented systematic analysis of patients’ anatomical reproducibility over multiple DIBH motion-management technique uses for pancreatic cancer RT. We used data from 20 patients; four DIBH scans were available for each patient to design 80 SBRT plans. Our results demonstrated that (i) there is considerable variation in OAR geometry and dose between same-subject DIBH scans; (ii) the RT plan designed for one scan may not be directly applicable to another scan; (iii) the RT treatment designed using a DIBH simulation CT results in different dosimetry in the DIBH treatment delivery; and (iv) this confirms the importance of adaptive radiation therapy (ART), such as MR-Linacs, for pancreatic RT delivery. The ART treatment delivery technique can account for anatomical variation between referenced and scheduled plans, and thus avoid toxicities of OARs because of anatomical variations between DIBH patient setups.

## 1. Introduction

Pancreatic cancer is a devastating disease; it is the fourth leading cause of cancer-related death and has less than a 10% overall survival (OS) rate 5 years after diagnosis [1]. One-third of diagnoses present with unresectable, locally advanced pancreatic cancer (LAPC) with significant risk for distant progression [2,3,4,5]. Distant progression may be delayed, and OS may increase, when local control is achieved using local-regional therapies such as RT. Only 30% of patients die of locally destructive pancreatic cancer [6]. Thus, it is of great importance to improve LC for LAPC patients [1,4,7,8,9,10,11,12,13,14,15].

Improved LC can be achieved using dose-escalated RT. Reaching a 70 Gy biological effective dose (BED) considerably enhances LC and overall survival rate at 2 years from 19 to 36%, and at 3 years from 9 to 31% [11,12,16,17,18,19,20,21,22]. However, dose escalation is severely hindered by the toxicity of adjacent organs at risk (OAR), namely the stomach, the bowel, and more prominently, the duodenum, as it wraps around the head of the pancreas (HOP), which accounts for 65% of pancreatic cancer diagnoses [23,24]. Geometrical uncertainties arise from delineation uncertainties, inter- and intra-fraction tumor motion, and anatomical variations [25]. To account for these uncertainties, error margins are often added to the gross tumor volume (GTV) to define the planning target volume (PTV). The PTV or the target volume thus typically overlaps OARs. As it is imperative to spare OARs, this directly limits RT plan optimization and hinders dose escalation [25,26]. 

Motion management techniques are typically used to reduce treatment planning margins [25,27,28,29,30,31]. Deep inspiration breath hold (DIBH) prevents respiratory-induced tumor motion by asking patients to hold their breath during treatment above a certain threshold that gates the radiation exposure [32]. DIBH effectiveness, however, is dependent on consistent tumor motion reproducibility within each breath hold (inter-breath-hold variation) [32,33]. This is because multiple breath holds are required for the delivery of one treatment fraction. In pancreatic cancer, inter-breath-hold variation arises from both the diaphragm and abutting abdominal soft tissue, which poses a unique challenge for tumor position reproducibility [27,33]. 

Previously, there have been three studies regarding DIBH pancreatic cancer tumor motion reproducibility. Murphy et al. reported the reproducibility of 2.5 mm for 3D pancreatic tumor position, but their study only included four patients [34]. Using 2D kV projection images from 19 patients, acquired at each beam angle, Teboh et al. showed that the intra-fraction DIBH variation was within 2 mm [35]. More recently, Han-Oh et al. performed a thorough investigation using 3D CT simulation and five cone beam CT (CBCT) of 20 patients to characterize intra-fraction tumor position variation, and reported an average treatment fiducial position variation of 1.2 ± 0.4 mm in the LR direction, 1.1 ± 0.4 mm in the AP direction, and 1.9 ± 1.0 mm in the SI direction. They concluded that inter-breath-hold variation is considerable, especially in the SI direction [32]; however, the dosimetric impact of these variations remained unexplored.

In this article, we present a detailed characterization of the DIBH variation effect on dosimetric indices and tumor–OAR relative geometry. The novelty of this study can be summarized as (i) using a unique dataset comprising four DIBH high-resolution CT scans for each of 20 pancreatic cancer patients; (ii) assessing the dosimetric impact of DIBH variation on the dose distribution and OAR dose; and (iii) quantifying 3D tumor–OAR geometrical variation using overlap-volume histogram (OVH) distances and evaluating the correlation between these anatomical measurements and OAR doses.

## 2. Materials and Methods

### 2.1. Data Collection

Data from 20 patients, under Johns Hopkins University IRB approval, were used for this study. As the standard of care, for each patient, one DIBH CT was acquired for treatment planning, in addition to three immediately acquired repeat DIBH CT sets for assessing the breath-hold ITV, making a total of four DIBH scans per patient. The CTs were acquired sequentially during CT simulation using a patient-specific fixed threshold active breath-hold coordinator. The first scan, taken immediately after venous contrast injection, was used as the reference for treatment planning, and the other three were used for inter-breath-hold variations assessment.

### 2.2. Radiotherapy Planning

A total of 80 SBRTs, using the volumetric modulated arc therapy (VMAT) technique, was designed. The patients’ original prescriptions were 33 Gy in 5 fractions. According to our institution’s RT planning protocol for pancreatic cancer, the plans were optimized to achieve 33 Gy in five fractions to 85% of the PTV. The initial treatment plans were generated using volumetric modulated arc therapy (VMAT), for which the PTV definition included breath-hold variation and a 2 mm setup margin. The beam arrangements included one or two full arcs per each patient’s breath-hold constraints. Pancreas, duodenum, and bowel objectives were 1 cc below 33 Gy and 20 cc below 20 Gy. Each plan was reviewed by our institution’s radiation oncology group and approved by the attending physician before delivery. Evaluation doses were generated using the RayStation treatment planning system (TPS; RaySearch Laboratories, AB, Stockholm, Sweden) by propagating the initial radiation fields onto co-registered breath-hold CTs acquired at the time of treatment simulation. 

### 2.3. OAR Dose Variability

We quantified geometric and dosimetric variability in OARs for both patient anatomy and dose distribution. First, we calculated the overlap volume histogram (OVH) distance ($L_{1cc}$, $L_{5cc}$ and $L_{20cc}$) for each DIBH CT set for the major pancreatic RT OARs (the duodenum, the stomach, and the bowel); $L_{1cc}$ is defined as the amount of uniform tumor expansion to have 1 cc overlap with an OAR, to quantify the 3D spatial geometric variation of OARs with respect to GTV. OVH distances are informative 3D physical measurements quantifying the spatial separation between the target and the OAR. Previous studies have shown that OVH distances are highly correlated with dosimetric indices; they have been used for predicting the OAR dose and automated and semi-automatic treatment planning [36,37,38,39,40]. 

Next, we measured and reported the V33Gy, V30Gy, and V20GY of each evaluation dose for the duodenum, the stomach, and the bowel; V33Gy is defined as the OAR volume receiving the 33 Gy dose or higher. Additionally, to better quantify the high-dose radiation received by OARs, we also measured the $D_{0.03cc}$ and $D_{2cc}$; $D_{0.03cc}$ is the minimum dose received by the most exposed 0.03 cc volume of the OAR. Finally, the correlation between OVH metrics and DVH indices (V33Gy, V30Gy, and V20Gy) was evaluated. 

### 2.4. Statistical Analysis

We quantified the between-subject variability by first calculating the average of each patient’s measurements as the representative value and then calculating the standard deviation of the mean values. Inter-breath-hold variability was calculated by first finding the standard deviation of each patient’s measurements; the average of these 20 standard deviation values was reported as the inter-breath-hold variability.

## 3. Results

### 3.1. Radiotherapy Planning Results

**Figure 1:**
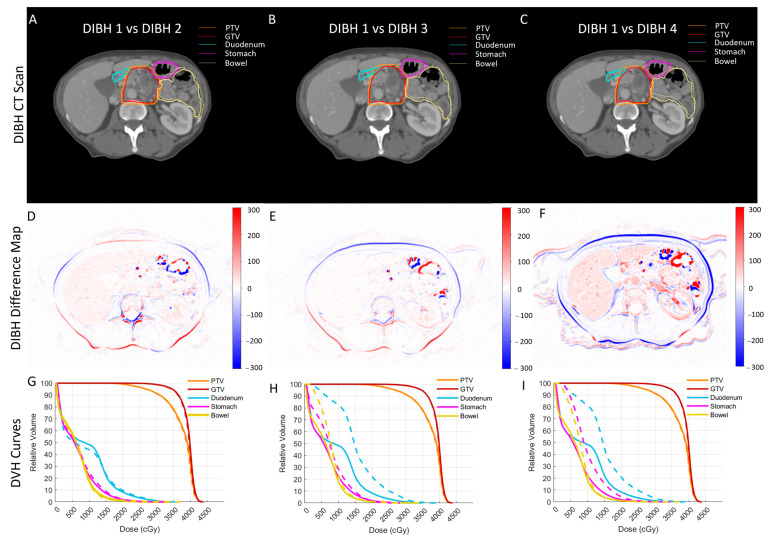
A typical case in which each column shows a pair including an initial DIBH scan used for patient treatment and another DIBH scan. (**A**–**C**) The initial CT and two sets of contours overlaid (solid for initial CT and dotted for DIBH CTs) to show the variability in contours’ and organs’ shapes. (**D**–**F**) The HU difference maps between the same pairs of scans to better demonstrate the organs’ shape variability. (**G**–**I**) The DVH comparison of dose distribution for the same pairs of breath-hold scans; even though the breath-hold levels were comparable and the same planning protocol was used, there is a noticeable difference between the dose distributions.

To avoid any planning bias, planning parameters, such as the number of beams and objective functions, were identical for each patient’s evaluation plan. RT plans were optimized to achieve 85% target coverage while maintaining clinically acceptable doses to OARs. We observed a noticeable inter-breath-hold variability in the regions of interest (ROI) contours. In Figure 1, we show a typical case where each column shows a pair including an initial DIBH scan used for patient treatment and other DIBH scans that were used to create the internal target volume (ITV). As seen, although the CT scans were acquired using the same protocol, there is a noticeable difference between the scans; more specifically, the OARs show the most difference.

Figure 1A–C show a typical initial CT scan of a patient with two sets of contours overlaid (solid and dotted lines); each column shows the initial CT vs. the second (A), third (B), and forth (C) DIBH CT contours. As seen, there is noticeable variability between organ shapes on each CT pair. This variability could potentially be caused by variability in the organ delineation process. Thus, to better demonstrate the inter-breath-hold variability in organ shape, we also created HU difference maps of initial CTs with each of the other DIBH CT scans (Figure 1D–F). The HU difference maps clearly show the variability in organ shapes more prominently seen in the organ boundaries. 

The inter-breath-hold variability in organ shape and corresponding contours led to variability in dosimetric indices. Figure 1G–I show the comparison of DVH curves for the same pair of CT scans and their dose distribution (solid lines for initial CTs and dashed lines for other DIBH scans). We included DVH curves for gross tumor volume (GTV), PTV, the duodenum, the bowel, and the stomach. We observed a noticeable difference between the OAR DVH curves, especially for the duodenum, due to its proximity to the tumor (head of the pancreas). 

### 3.2. Variability Analysis

**Figure 2:**
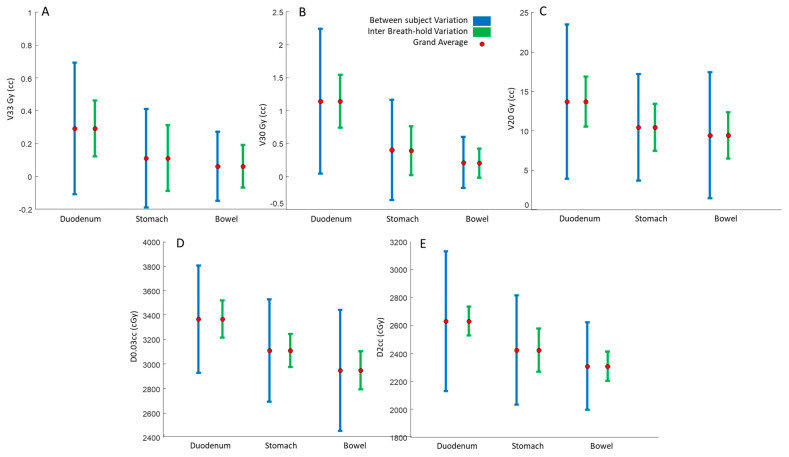
Illustration of between-subject (blue) and within-patient (green) breath-hold variations for (**A**) V33Gy, (**B**) V30Gy, (**C**) V20Gy, (**D**) *D*_2cc_, and (**E**) *D*_0.03cc_.

Similar to our qualitative results (in Figure 1), our quantitative results also demonstrated that there was considerable variability between OAR doses for each patient (Figure 2, blue error bars). This between-subject variability was directly related to patient-specific anatomical differences. More importantly, we found that the OAR dose was also highly variable between the four DIBH scans of each patient. This inter-breath-hold variation is shown in Figure 2’s green error bars. 

To better quantify the variability, we reported the coefficient of variation (CV) for inter-breath-holds V33Gy, V30Gy, and V20Gy. The averaged CVs for the duodenum V33Gy, V30Gy, and V20Gy were 67%, 38%, and 15%, respectively; for the stomach they were 121%, 73%, and 16%, respectively, and for the bowel they were 119%, 86%, and 27%, respectively. The average normalized deviations from the mean value (CV) of *D*_2cc_ and *D*_0.03cc_ for the duodenum were 3.9% and 3.9%, respectively; for the stomach they were 4.8% and 3.4%, respectively, and for the bowel they were 3.3%, and 4.1%, respectively. 

We used OVH distances to quantify the 3D relative geometry of tumors and OARs. Our repeated measures analysis showed that $L_{1cc}$, $L_{5cc}$, and $L_{20cc}$ OVH distances had a grand average, between subject and inter breath-hold variations (mm), of (2.5, 3.87, 1.8), (4.7, 4.76, 2.83), and (9.9, 7.17, 5.5), respectively, for the duodenum, (4.1, 3.2, 3.88), (6.64, 4.09, 4.27), and (11.23, 4.9, 6.9), respectively, for the stomach, and (4.58, 3.68, 3.21), (7.13, 3.8, 4.3), and (11.3, 4.38, 6.73), respectively, for the bowel.

### 3.3. OVH–Dose Correlation Analysis

Previously, it has been shown that OVH distances have a high correlation with dosimetric indices. Our results showed that OVH $L_{1cc}$, $L_{5cc}$, and $L_{20cc}$ had a high correlation with duodenal high and mid-dose volume (V33Gy, V30Gy, and V20Gy). We found that OVH distances had an inverse power correlation with dose indices defined as

$$y=x^{a}\;where\;a<0$$

The power relation was inspired by the fact that OVH distances are of a distance (r) or 1D nature, while V33Gy and V20Gy are of a volumetric ($r^{3}$) or cubic 3D nature. As alternatives, we used both sigmoid and Gaussian functions; however, in our experience, the power relationship showed the highest correlation.

**Figure 3:**
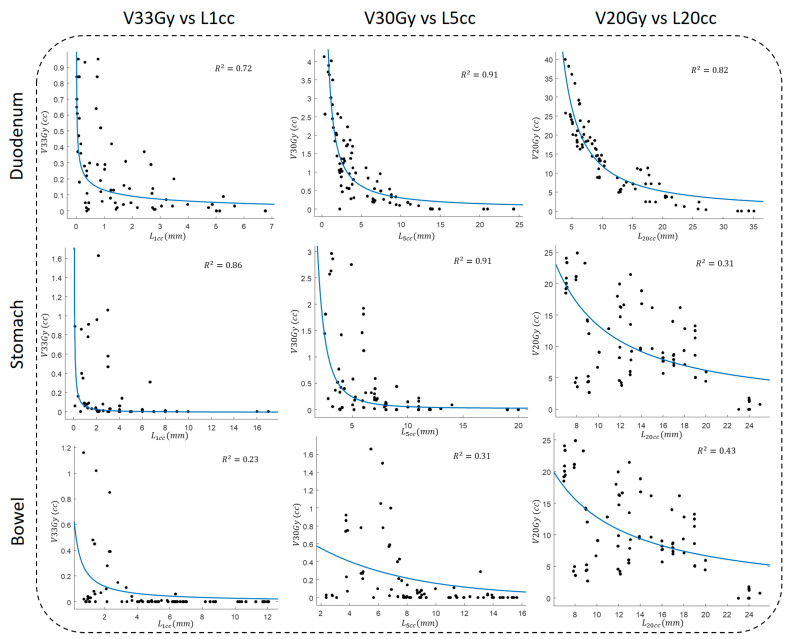
The fitting results for adjacent OARs between V33Gy, V30Gy, and V20Gy and $L_{1cc}$, $L_{5cc}$, and $L_{20cc}$ OVH distances, respectively.

Figure 3 shows scatter plots of dose volumes against corresponding OVH distances, along with fitted curves and corresponding $R^{2}s$. Our results suggest a strong correlation between duodenal dose volume and OVH distance. More specifically, in agreement with our previous study [41], we observed the highest correlation between the duodenal volume dose and the OVH distance; every two-fold increase in $L_{1cc}$, $L_{5cc}$, and $L_{20cc}$ resulted in a 20%, 13%, and 40% reduction in V33Gy, V30Gy, and V20Gy, respectively.

## 4. Discussion

We used unique data comprising four DIBH high-resolution CT scans for each of 20 pancreatic cancer patients to perform a detailed analysis of inter-breath-hold anatomical variation and its effect on dosimetric indices. Previous studies, such as those of Lens et al. and Han Oh et al., found considerable inter-fraction position variation in pancreatic tumors, as well as intra-fraction position variation, which may not be compensated for by DIBH [32,42]. We also found a high intra-fraction anatomical variability; however, one major strength of our study was using high-resolution CTs. Unlike CBCT or KV projections, these CTs showed a good soft tissue contrast that reduced the effect of contouring uncertainty and variation on our results; thus, the reported variation was due to anatomy-related variations. In practice, it is assumed that there is minimal inter-breath-hold variation in OAR volume and positioning in relation to the PTV between simulation and treatment delivery; thus, conventionally, a plan designed based on a single breath-hold simulation CT is used for delivery. Our results support the importance of ART to account for these anatomical variations between the reference plan and the scheduled plan, even though the patient is under the same DIBH motion management technique during both imaging and treatment delivery.

**Figure 4:**
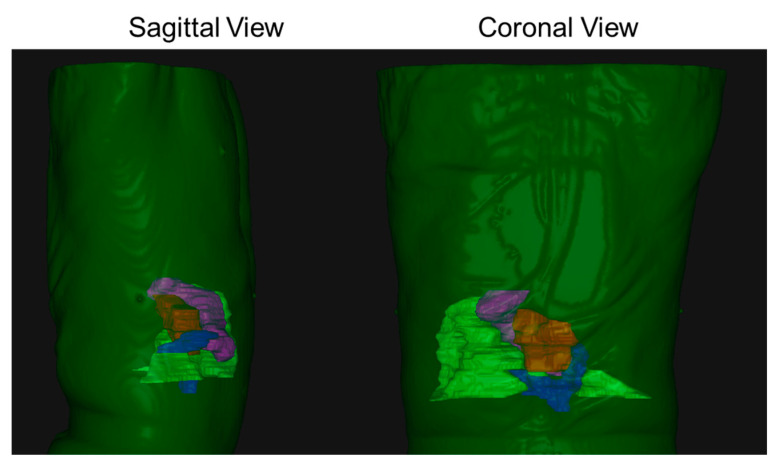
A 3D rendering of a typical patient body (dark green shade) with a tumor (red shade), the duodenum (blue shade), the stomach (magenta shade), and the bowel (light green shade). As seen from both sagittal and coronal views, the tumor–duodenum assembly is situated for the most part in the SI plane.

In this paper, we present a detailed novel analysis of tumor–OAR relative geometric variations, as opposed to previous studies that have mostly focused on tumor position inter-fraction and intra-fraction variability. Jayachandran et al. analyzed the shift in location of fiducials with respect to the bony structures and reported mean shifts of 1.8, 1.6, and 4.1 mm (left-right (LR), anterior-posterior (AP), and superior-inferior (SI), respectively) [43]. Another comparable study of 19 pancreatic cancer patients treated using DIBH SBRT found mean intra-fraction shifts of 1.5, 2.0, and 3.0 mm [35]. Hill et al. characterized mean intra-fraction shift across 150 intra-fraction CBCTs, and reported average shifts of 2.0, 2.0, and 2.3 mm [33]. As seen in Figure 2, the duodenum shows a large variability. This is because of the anatomy of the duodenum and the head of the pancreas; the duodenum has a “C-loop” shaped structure that wraps around the tumor, and this whole assembly is situated in the SI plane. To better illustrate our point, we show a 3D rendering of a typical patient body (dark green shade) with a tumor (red shade), the duodenum (blue shade), the stomach (magenta shade), and the bowel (light green shade) in Figure 4. Moreover, due to the proximity of the duodenum, it has a large impact on the dosimetric indices as well.

Figure 1 shows that the intestines have the maximum variability, as indicated by the highest values in HU difference maps (D–F); their geometry relative to the tumor changes with each breath-hold. This implies that during online ART, it is important to track not only the tumor, but also its relationship with surrounding OARs. In this case, metrics, such as OVH, can be useful in providing 3D quantitative feedback; as they have a high correlation with the receiving dose, they can be used to create predictive dose models and more effective ART delivery. Our results showed that an increase in OVH distances, which is synonymous with the structures being further away from each other, leads to an OAR dose reduction. More specifically, we found a strong inverse power relationship between duodenal OVH distances and dose volume; every two-fold increase in *L*_1cc_, *L*_5cc_, and *L*_20cc_ resulted in a 20%, 13%, and 40% reduction in V33Gy, V30Gy, and V20Gy, respectively. In agreement with our previous findings [41,44], this demonstrates the potential benefits of using a duodenal spacer [21,45,46,47] to increase the duodenum–tumor separation, and thus enable a safe and effective dose escalation.

The average inter-breath-hold *L*_1cc_ OVH variation was 1.8 mm for the duodenum, 3.88 mm for the stomach, and 3.21 mm for the bowel. As seen in Figure 3, the *L*_1cc_ had a high correlation with high radiation dose volume (V33Gy); consequently, given our results, nearly 4 mm expansion from the original tumor was needed to account for 3D tumor–OAR geometric variation between the four DIBH scans. Such a large planning margin results in a large overlap of PTV with OARs that greatly affects the plan optimization and hinders dose escalation to avoid OAR toxicity. Daily MR-guided ART (MRgART) has many advantages over traditional RT planning, including the ability to conform a high-dose radiation cloud to anatomical changes and tumor movement [48,49], which can potentially tighten the margins and increase the therapeutic effectiveness and safety of RT treatment [50,51]. Moreover, it can enable safe dose escalation, which pancreatic cancer can greatly benefit from [16,52,53]. With excellent soft-tissue contrast and online plan adaptation, MRgART could resolve the toxicity-related challenges of dose escalation [20,54,55]. Previously, it was shown that MRgART can reduce irradiated liver volumes without an ITV [56,57,58,59], and that tighter PTV margins can be achieved to ensure sparing nearby radiosensitive OARs while achieving target doses [56,57,58,59]. Our results also imply that MRgART for pancreatic cancer can (i) tighten the PTV margin and thus enable safe dose escalation, and (ii) remove the need for multiple breath-hold scans and shorten the RT treatment workflow.

Finally, we are aware that our study may have a few limitations. First, at this stage we quantified the dosimetric impact of inter-breath-hold variation in OAR positioning using CT simulation. The variation might be different fraction by fraction, depending on breath-hold technique changes between the chest and diaphragmatic breathing, daily stomach filling, or gas patterns. The future step is to understand the correlation of these factors with inter-breath-hold variation and explore a mitigation method. Another limitation of our study was that although we believe that ART techniques, such as MRgART, can address these variabilities and reduce their adverse effect on OAR toxicities, in this current study we did not demonstrate the aforementioned benefits of ART for inter-breath-hold variations. This is because at the moment our institution is not equipped with ART delivery machines. As part of our future study, we will demonstrate the effectiveness of ART to address these variabilities. 

## 5. Conclusions

In this study, we used data from 20 pancreatic cancer patients, who each had four separate DIBH CT scans, to perform a detailed analysis of inter-breath-hold anatomical variation. We assessed the dosimetric impact of DIBH variation on dose distribution and the OAR dose and quantified the 3D tumor–OAR geometrical variation using OVH distances. Our results showed that there are considerable anatomical variations in inter-breath-hold scans. We found a high correlation between the OAR dose and OVH distances; an increase in OVH distances led to a considerable decrease in the OAR dose. We also observed that inter-breath-hold anatomical variations directly affected the OAR dose, which raises the concern that the standard of care single DIBH CT practice for multi-fraction treatment regimens may be suboptimal. We believe that pancreatic cancer RT treatment can greatly benefit from MRgART to account for anatomical variation and enable dose escalation.

## Data Availability

Raw data supporting the conclusions of this article will be made available by the authors upon request.
